# Successful utilization of angioembolization and delayed laparoscopy in the management of grade 5 hepatic laceration: Case report and literature review

**DOI:** 10.1016/j.ijscr.2019.05.011

**Published:** 2019-05-10

**Authors:** Adel Elkbuli, John D. Ehrhardt, Mark McKenney, Dessy Boneva

**Affiliations:** a Department of Surgery, Kendall Regional Medical Center, Miami, FL, United States; b University of South Florida, Tampa, FL, United States

**Keywords:** High-grade hepatic laceration, Angioembolization, Solid organ injury grading, Delayed laparoscopy, Hemoperitoneum, Biliary peritonitis

## Abstract

**Introduction:**

The liver is the most commonly injured solid organ in blunt abdominal trauma. Although the incidence of hepatic lacerations continues to rise, non-operative management with angioembolization is currently the standard of care. While active arterial hemorrhage is commonly embolized in grade 3 or 4 injuries, patients with grade 5 injuries frequently require operative intervention.

**Presentation of case:**

A 30-year-old man presented to our level I trauma center following a motor scooter accident. CT abdominal imaging revealed a grade 5 right lobar hepatic laceration. He underwent successful angioembolization without further hemorrhage. The patient later developed abdominal discomfort that worsened to peritonitis and he was taken for laparoscopic drainage of massive hemoperitoneum with bile peritonitis. Postoperatively, the patient’s abdominal pain abated and he tolerated oral dietary advancement.

**Discussion:**

Surgical management of blunt hepatic trauma continues to evolve in tandem with minimally invasive interventional techniques. Patients with high-grade lacerations are at higher risk for developing biliary peritonitis, hemobilia, persistent hemoperitoneum, and venous hemorrhage after angioembolization. Accordingly, the primary role of surgery has shifted in select patients from laparotomy to delayed laparoscopy to address the aforementioned complications.

**Conclusion:**

While laparotomy remains crucial for hemodynamically unstable patients, angioembolization is the primary treatment option for stable patients with hemorrhage from liver trauma. The combination of angioembolization and delayed laparoscopy may be considered in stable patients with even the highest liver injury grades.

## Highlights

• We present a patient who sustained a grade 5 hepatic injury with active arterial extravasation on CT, which was successfully angioembolized.
• Delayed diagnostic/therapeutic laparoscopy should be considered in the management algorithm of high grade liver injuries.

## 1 Introduction

Hepatic injury is the most common injury in blunt abdominal trauma. The incidence and severity of hepatic lacerations continues to rise and positively correlates with motor vehicle collision trends. Hepatic trauma exists on a spectrum from small capsular tears to severe parenchymal lesions that may be complicated by retrohepatic vena cava or portal triad involvement. The American Association for the Surgery of Trauma (AAST) initially established a grading system for hepatic lacerations in 1989 where low-grade lesions (1, 2) and high-grade (3–5) lesions were organized according to severity and risk of death. This standardized grading system (Table 1) was updated most recently in 2018 and provides framework to guide management strategies in hepatic trauma [1].

**Table 1:** American Association for the Surgery of Trauma 2018 update on hepatic injury grading.

AAST Liver Injury Scale—2018 Update^1^	
Grade	Associated Findings
1	• Subcapsular hematoma <10% surface area • Parenchymal laceration <1 cm in depth • Capsular tear identified intraoperatively
2	• Subcapsular hematoma 10–50% surface area • Intraparenchymal hemorrhage <10 cm in diameter • Laceration 1–3 cm in depth
3	• Subcapsular hematoma >50% surface area • Ruptured subcapsular or parenchymal hematoma • Intraparenchymal hemorrhage >10 cm in diameter • Laceration >3 cm in depth
4	• 25–75% lobar parenchymal disruption • Active hepatic hemorrhage with extension into the peritoneum
5	• >75% lobar parenchymal disruption • Juxtahepatic venous injury involving retrohepatic vena cava and central major hepatic veins

1 Low grade hepatic injury.

Advances in interventional radiology and critical care medicine have shifted the standard of care towards non-operative management, allowing for fewer unnecessary laparotomies and improved outcomes in patients with hepatic lacerations [[2], [3], [4]]. Hemodynamically stable patients with active arterial hemorrhage can undergo diagnostic angiography and therapeutic angioembolization to manage their injuries. Following successful angioembolization, patients with minimal biloma and hemoperitoneum can often recover without further intervention. Patients with high-grade lacerations complicated by significant hemoperitoneum can undergo angioembolization when hemodynamically stable, but up to two-thirds ultimately require laparotomy to control recurrent hemorrhage or biliary leaks [5].

Herein, we present a case of a grade 5 hepatic laceration where hemorrhage was initially managed with angioembolization. Laparoscopy was utilized four days later for worsening abdominal pain from hemoperitoneum with bile peritonitis. This case is reported with consideration to the SCARE criteria [6].

## 2 Presentation of case

**Fig. 1:**
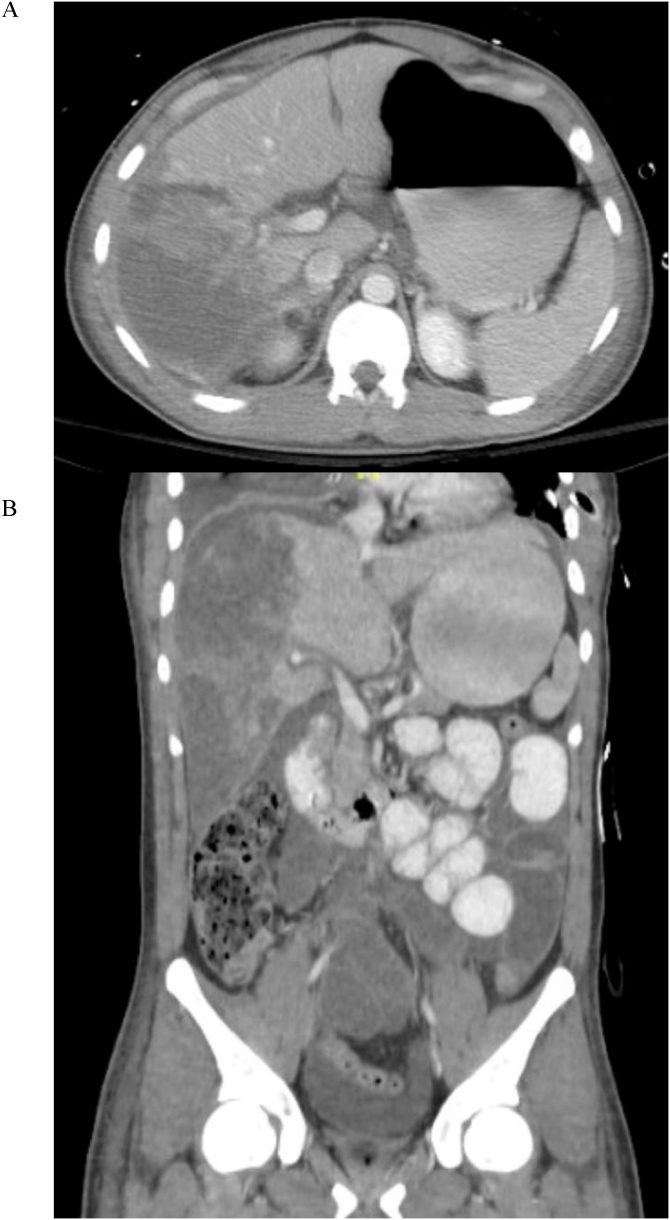
Axial (A) and coronal (B) abdominal CT scans demonstrating AAST grade 5 hepatic laceration with extravasation in segments 5 and 6.

A 30-year-old man presented to the trauma resuscitation arena after a motor scooter collision in which he was an un-helmeted scooter driver who was hit by a car. His vital signs on arrival were significant for a blood pressure of 90/60 mmHg, which improved with intravenous fluids per Advanced Trauma Life Support (ATLS) protocol. Ultrasonography demonstrated free fluid in the abdomen. Once hemodynamically stable he underwent abdominal CT imaging, which was significant for a grade 5 right lobe hepatic laceration with active arterial extravasation from Couinaud segments 5 and 6 (Fig. 1).

**Fig. 2:**
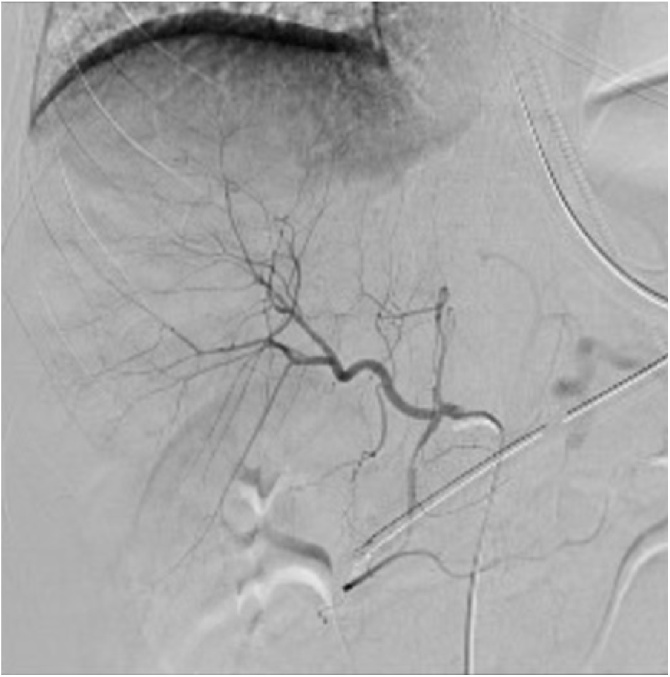
Hepatic angiogram demonstrating contrast blush from active right hepatic arterial branch hemorrhage.

Interventional radiology was consulted because of the extent of injury and active hemorrhage demonstrated on CT. Interventional radiology expeditiously performed hepatic visceral arteriography and selectively embolized an actively bleeding branch of the right hepatic artery. (Fig. 2). During the embolization, the patient received two units of blood and his hemoglobin remained stable post-procedure without further transfusion. Over the next four days, his total bilirubin peaked at 2.4 mg/dL, and he developed increasing abdominal pain which progressed to rebound tenderness. Repeat abdominal CT on the fourth hospital day suggested a viable liver with sizable parenchymal fluid collection. He was taken to the operating theater for diagnostic and possibly therapeutic laparoscopy. Preoperative diagnoses were bile peritonitis and hemoperitoneum.

Intraoperatively, the liver was non-bleeding with healing stellate lacerations and considered viable. Suction evacuated approximately four liters of bile mixed with hematoma from the peritoneum. Following this, no active biliary leak or hemorrhage was seen. Postoperatively, his abdominal pain resolved and he tolerated dietary advancement. He recovered without further complications and was discharged on the seventh postoperative day after a total 11-day hospitalization. At four-week outpatient follow up, the patient had no injury related complaints and normal hepatic function per laboratory.

## 3 Discussion

Many patients with moderate-grade hepatic lacerations undergo angioembolization for hemorrhage control as definitive treatment, whereas high-grade injuries can require laparotomy to achieve hemostasis [5]. We presented a case of a grade 5 hepatic laceration where hemorrhage was successfully managed with angioembolization. However, our patient developed progressively worsening peritoneal signs after the angioembolization. Although acute abdomen is a common indication for laparotomy, our patient’s hemodynamic and hematologic stability after embolization influenced the decision for a laparoscopic approach.

Interventional embolization techniques have allowed many patients with moderate-grade hepatic lacerations avoid laparotomy. Nonetheless, there are complications after angioembolization which may require operative intervention, namely bile peritonitis, missed enteric injury, unresolved hemoperitoneum, and ongoing hepatic venous or portal venous or recurrent hepatic arterial hemorrhage [7]. Less common complications include biliary fistula [8] and hepatic artery pseudoaneurysm [9]. Recovering patients can manifest clinical and laboratory signs of systemic inflammation through fever, tachycardia, leukocytosis, and peritonitis, all of which are often accompanied with radiologic evidence of peri-hepatic fluid collection. For these patients, delayed surgical intervention with diagnostic laparoscopy and washout is safe regardless of the extent of hepatic injury and often dramatically improves peritonitis with little associated morbidity [10]. On laparoscopic investigation, it is uncommon to encounter active hemorrhage that entails conversion to laparotomy.

Surgeons at the University of Louisville were early advocates for delayed laparoscopy after angioembolization in blunt hepatic trauma [[11], [12], [13], [14]]. They outlined their operative technique in a case series with fifteen patients. Preferred patient positioning with 5° right table tilt and moderate Trendelenburg allows fluid to accumulate in the right upper quadrant. Interestingly, they cautioned against interrogating the liver to assess the extent of hepatic injury to avoid dislodging a nascent clot and triggering repeat hemorrhage. Nonetheless, they emphasized the importance of carefully examining the rest of the abdominal cavity for occult injuries [11].

Some patients develop down trending hemodynamic parameters and raise clinical suspicion for ongoing venous hemorrhage. Laparotomy with perihepatic packing is the traditional mainstay [15], but a number of modern hemostatic agents may allow for laparoscopic management of posttraumatic venous hemorrhage. A 2017 animal model study demonstrated that biologic fibrin dressings are effective in managing hemorrhage from penetrating hepatic injuries [16]. An additional report described the use of a surgical adhesive composed of bovine serum albumin and glutaraldehyde (BioGlue^®^ Cryolife, Kennesaw, GA) to halt biliary leakage following blunt hepatic trauma [17]. While they used the adhesive during laparotomy, this application has potential for laparoscopic delivery.

Uncommonly, major hepatic trauma can necessitate resection. Although resection is radical and traditionally reserved for laparotomy, a growing number of randomized studies on non-trauma patients now discuss laparoscopic liver resection. In the setting of trauma, there is currently one case report where laparoscopic left lobar resection was performed five days after a grade 3 laceration [18].

Follow-up CT imaging is a common practice to ensure that healing progresses without the development of necrotic hepatic segments. Many surgeons advocate for repeat CT imaging, especially in the context of a high-grade laceration. Nonetheless, a 2005 retrospective study of 530 patients with blunt hepatic injury concluded that follow up CT imaging may be unnecessary for some cases [19]. The subset of patients in their study with grade 5 lacerations (n = 15) showed improved or unchanged hepatic tissue on follow-up CT within one week of injury, with no patients showing worsened CT findings. We counseled our patient for repeat abdominal CT, but he declined because he had no symptoms and was concerned for additional radiation. Despite not obtaining a follow-up CT, we felt assured by his outpatient clinic examination showing normal abdominal exam and normalized hepatic function laboratories.

## 4 Conclusion

We present a case of a 30-year-old man who sustained a grade 5 right lobar hepatic laceration following a motor vehicle collision. Although angioembolization successfully stopped an active right hepatic artery hemorrhage, he developed peritonitis and underwent delayed laparoscopic investigation on the fourth admission day. Following evacuation of hemoperitoneum and biloma with peritoneal lavage, our patient made an expedient recovery.

This case highlights an excellent outcome with angioembolization and delayed laparoscopy in a grade 5 hepatic laceration. Recent literature suggests that up to two-thirds of these severe parenchymal injuries require laparotomy to address active hemorrhage. The primary role of surgical care in stable patients is now concerned with managing sequelae from angioembolization, most commonly bile peritonitis and retained hemoperitoneum. Combined angioembolization and delayed laparoscopy can be considered in stable patients regardless of the AAST liver injury grade.

Laparotomy with perihepatic packing for slow and persistent venous hemorrhage may also evolve in the future with the success of biologic pro-thrombic agents and surgical adhesives. As the utility of laparoscopy continues to grow in blunt hepatic trauma, some patients requiring resection may benefit from a laparoscopic approach, which has recently entered the literature.

## Conflicts of interest

None.

## Sources of funding

None.

## Ethical approval

This report was conducted in compliance with ethical standards.

## Consent

Informed written consent has been obtained and all identifying information is omitted.

## Author’s contribution

Adel Elkbuli, Dessy Boneva, Mark McKenney – Conception of study, acquisition of data, analysis and interpretation of data.

Adel Elkbuli, Dessy Boneva, John D. Ehrhardt Jr. – Drafting the article.

Dessy Boneva, Mark McKenney – Management of case.

Adel Elkbuli, John D. Ehrhardt Jr., Dessy Boneva, Mark McKenney – Critical revision of article and final approval of the version to be submitted.

## Registration of research studies

This is a case report study.

## Guarantor

Dessy Boneva.

Mark McKenney.

## Provenance and peer review

Not commissioned, externally peer-reviewed.
